# Prevalence and associated risk factors of hypertension among university staff: a cross-sectional study at the University of Juba, South Sudan

**DOI:** 10.11604/pamj.2025.51.76.47018

**Published:** 2025-07-18

**Authors:** Kon Paul Alier, Oromo Francis Omojo, Akway Medho Cham, Ezbon WApary, Kenneth Lado Lino Sube, Joseph Lako, Achol Madhieu Wol, Josephine Agidong, Zechariah Yere Gib, Benedict Bol Makur, Nyariak Malei Ayuel

**Affiliations:** 1 School of Medicine, University of Juba, Juba, South Sudan,; 2 School of Applied and Industrial Science, University of Juba, Juba, South Sudan

**Keywords:** Hypertension, prevalence, University of Juba, staff, South Sudan

## Abstract

**Introduction:**

hypertension refers to an average systolic blood pressure greater than or equal to 140 mmHg and/or diastolic blood pressure greater than or equal to 90 mmHg. In South Sudan, published evidence on the disease is scarce. This study aimed to assess the prevalence and associated risk factors of hypertension among the University of Juba staff.

**Methods:**

a cross-sectional study among 409 University of Juba staff was conducted. Data collection used a structured questionnaire and blood pressure measurements and body weight were made using a Littman Stethoscope Classic III, mercurial sphygmomanometer and weighing scale fitted with a stadiometer, respectively. Sample was estimated using Cochran´s formula. Institutional ethical clearance was obtained from the College of Medicine and the University of Juba Directorate of Research and Publication. Permission was also obtained from each college and informed consent from each respondent. The software Epidata Manager 4.6.0.6 and IBM SPSS 23.0 were used for data entry and analysis respectively. Descriptive statistics, chi squared tests and multivariate logistic regression analysis were performed to explore factors associated with hypertension.

**Results:**

out of 409 participants, the majority were aged 28-37 (n=147) years, more than two thirds (n=260) were males, the prevalence of hypertension was 24%. At 95% confidence interval, significantly associated risk factors were age (aOR: 5.86, 95% CI: 2.51-13.70; P<0.001), alcohol consumption (AOR: 2.20, 95% CI: 1.16-4.17; P=0.016) and body mass index ≥ 25 (AOR: 2.42, 95% CI: 1.41-4.14; P<0.001).

**Conclusion:**

close to a quarter of the University of Juba staff is hypertensive. Age, alcohol consumption and increased body mass index were significantly associated with the disease. The study recommends tailored preventive interventions against hypertension at the university.

## Introduction

Hypertension refers to average systolic blood pressure (BP) greater than or equal to 140 mmHg and/or diastolic BP greater than or equal to 90 mmHg [1]. Its increase worldwide is due to an increase in the aging population as well as exposure to lifestyle risk factors consisting of unhealthy diets and lack of physical activity [1]. The disease is increasingly becoming an important medical and public health issue as its prevention and management pose major public health challenges [2,3]. Globally, 1.28 billion adults aged 30 - 79 years have hypertension and two-thirds of these are found in low- and middle-income countries [4]. There is evidence that the increasing proportion of illiteracy, limited access to healthcare services and facilities, unhealthy eating habits, poverty, and expensive cost of prescribed antihypertensive medications, and regional variations in hypertension risk factors, such as obesity, alcohol consumption, unhealthy diet and lack of physical activity, likely contribute to these regional differences [1,5,6]. For instance, in China and Sweden, a study conducted among men and women showed men had a higher prevalence of hypertension (43% in Sweden, 39% in China) while their female counterparts had 29% and 36% respectively [7]. In Saudi Arabia, a study on the demographic, behavioral, and hypertension risk factors found that 30.3% of respondents were hypertensive and 69.4% of all respondents had low physical activity, 49.6% were obese, 34.4% unhealthy diet, 32.1% had dyslipidaemia, and 25.1% had history of diabetes, 12.2% were found to be smokers, 15.4% self-reported feeling sad with 16.9% having a history of periods of stress and 6.8% had persistent stress while another third had a low educational level [8]. In Chile the prevalence of hypertension was at 30.8%, a drop from 32.0 and 34.0 in 2010 and 2003 respectively [9].

In a survey in 7 communities in four African countries (Kenya, Nigeria, Tanzania and Uganda) the prevalence of hypertension was 25.4% [10] while a facility-based study among adult patients in northwest Ethiopia found a higher prevalence, at 44.91% [11]. Even within Ethiopia, studies report different findings as shown by a similar study among workers at Hawassa University which found a prevalence of 19.7% [12]. A sub-Saharan Africa (SSA) multi country study that examined hypertension among different population groups found an overall prevalence of 25.9% but varying prevalence among different population groups; 25.8% for nurses, 23.2% for school teachers, 20.5% for peri-urban dwellers and lowest among rural residents (8.7%) [13]. The study established the risk factors as population group, old age, increased body mass index (BMI), high fasting plasma glucose, low level of education and tobacco use [2]. A Study from Sudan, from where South Sudan separated in 2011, showed disparities in prevalence of hypertension ranging from 15% to 50% [13]. This is most probably due to regional and ethnic variations. In countries undergoing epidemiological transition like South Sudan, the burden of hypertension is consistently increasing. There are few studies conducted in the country one of which is a pioneer retrospective cohort study among potential blood donors at Juba Teaching Hospital which reported a prevalence of 19.3% [14]. Another study conducted at Malakia Diabetic Centre for screening patients with diabetes for diabetic retinopathy showed the proportion of known patients with hypertension at 48.1% and those with high BP at 43.5% [15]. Apart from these studies, data on hypertension in South Sudan remain scarce. Without appropriate knowledge of the disease prevalence and the associated risk factors, it is difficult to confront and bring it under control. Therefore, this study aimed to assess the prevalence and associated risk factors of hypertension among the University of Juba staff.

## Methods

**Study design and setting:** the study adapted a cross-sectional design where primary quantitative data were collected, analysed and reported. It was conducted at the University of Juba, a national university located at two campuses, Atlabara (main), and Customs Area. Founded in 1975 in Juba City, the university hosts a student population of 40,000 spread across 21 schools, 2 colleges, 3 institutes, and 4 specialized centres. The university employs 2,274 academic and non-academic staff who were the target population of this study [16].

**Study population:** this study included 409 teaching and non-teaching staff of the University of Juba who provided voluntary informed consent and who were at least 18 years old. Exclusion criteria included being a non-staff of the University of Juba, minor and refusing to consent.

**Data collection:** each participant was interviewed by taking personal details and two BP measurements were taken; the first measurement was taken at the beginning of the interview and the second one at the end. The average measurement was then calculated by adding the two measurements and dividing the result by two. The questionnaire had provisions for recording measurements. Sample size was calculated using Cochran´s formula for estimating finite populations which considered a variance of 50% at 95% confidence interval [17]. The adjusted minimum sample was 361 but the team surpassed that and collected data from 409 participants. All schools, colleges, centers, institutes and the general university administration were given an equal chance of participation but sampling at individual level was done conveniently; any member of staff found in the office or school at the time of data collection and consented was interviewed. Measurement of BP and weight was done using mercurial sphygmomanometer, desk type, Littman Stethoscope Classic III and a weighing scale fitted with a stadiometer respectively. Weight was measured to the nearest 0.1 kilogram, while height was recorded to the nearest 0.1 centimeter. Body mass index was calculated by dividing the weight in kilograms by the square of height in meters, and is reported in kilograms per square meter.

**Definitions:** hypertension defined by average BP greater than or equal to 140/90 mmHg. Prevalence of hypertension was obtained by dividing the number of respondents with average BP greater than or equal to 140/90 mm Hg by the total of respondents. Body mass index was obtained by dividing the weight in kilograms by the square of height in meters and recorded as kilograms per square meter.

**Statistical analysis:** the software Epidata Manager 4.6.0.6 and IBM SPSS 23.0 were used for data entry and analysis respectively. Descriptive statistics were obtained and presented. Pearson Chi-squared tests were performed to predict associated risk factors. At the 95% confidence limit, p-values less than or equal to .05 were considered significant. The significant factors were entered into multivariable logistic regression analysis, of which crude odds ratios (OR) and adjusted odds ratios (aOR) were obtained to understand the extent to which the significant risk factors accounted for change in hypertensive status among the respondents.

**Ethical considerations:** the study followed all ethical principles in the Declaration of Helsinki. Institutional ethical clearance was obtained from the School of Medicine Ethics Committee (letter dated 11/9/2023 with reference No. 5/2023). The University Directorate of Research and Publication also cleared the study. Informed consent was obtained from each school and participant. Additional safeguards were ensured throughout the research period; participants´ information was kept confidential and anonymous in order to ensure privacy by storing the data in a password-protected file only accessible to the research team. Similarly, participation was voluntary; there was no coercion, intimidation of any kind and no penalty for non-participation or withdrawal from the study. There was no conflicting interest in this study.

## Results

**Figure 1:**
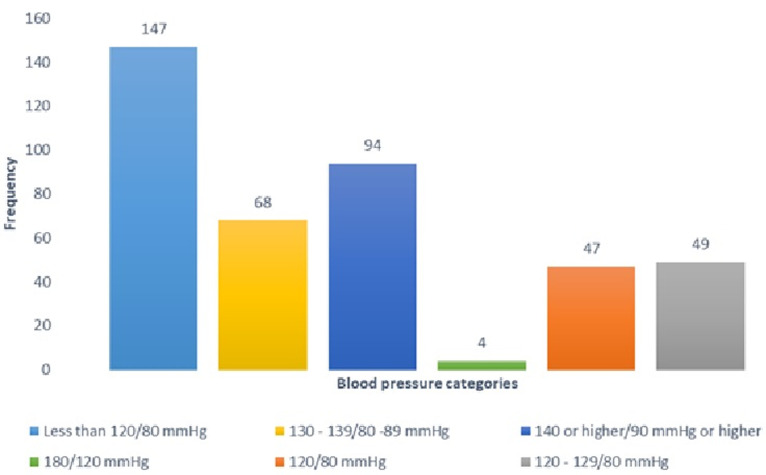
respondents' average blood pressure status

Table 1 presents the general characteristics of the participants. Out of 409 respondents, the majority were aged 28-37 (n=147) years, and more than 50% (n=249) were aged 28-47 years, and just about 3% were aged 67 (n=13) or older. More than two-thirds (n=260) were males, and 78.5% (n=321) were married, while more than a third (n=130) had earned at least a master´s degree. Most of the respondents (n=171) were academic staff and 96.8% (n=396) lived in the city. More than ninety-three percent (n=383) said they were non-smokers while 83.1% (n=340) were not drinking alcohol. However, one third (33.3%) (n=23) of those who admitted taking alcohol had hypertension. Just over 30.1% (n=123) exercise occasionally and more than half (n=208) were not aware of their BP status, although 56.2% (n=230) had at least screened for the disease at some point in time. More than 45% (n=187) said they consume more than 2 teaspoons per day. More than 68% (n=280) had no family history of hypertension and 76% (n=311) had no family history of diabetes. Sixty-three percent (n=259) of the respondents knew what hypertension is and 58% (n=237) could define diabetes correctly. In terms of BMI, 52.5% (n=215) were either overweight (n=147) or obese (n=68). Respondents whose average BP was equal to or greater than 140/90 mm Hg were considered hypertensive and these constituted 24% (n=98) while those in the pre-hypertensive stage constituted 28.6% (n=117) (Figure 1). At 95% confidence interval, bivariate analysis shows significantly associated risk factors as age (X^2^(5) =42.57, P<.001), marital status (X^2^(3) = 12.08, P<0.001), alcohol consumption (X^2^(1) =4.00, P=0.049), diabetic (X^2^(1) =10.98, P<.001) and increased body mass index (X^2^(3) =23.25, P<.001). However, from the regression model, only age, alcohol consumption and increased BMI were significant factors; a university staff aged 38-57 years was 5.9 times more likely to be hypertensive (AOR: 5.86, 95% CI: 2.51-13.70; P<0.001), those who consumed alcohol were two times more likely to be hypertensive compared to non-consumers (AOR: 2.20, 95% CI: 1.16-4.17; P=0.016). Similarly, respondents with BMI^2^ 25 were more likely to be hypertensive (AOR: 2.42, 95% CI: 1.41-4.14; P<0.001) Table 2.

**Table 1:** general characteristics of the respondents

Variables ( n = 409)		Hypertensive status?		Total n (%)
		Yes n (%)	No n (%)	
Age in years	18-27	0 (0)	12 (100)	12 (2.9)
	28-37	13 (8.8)	134 (91.2)	147 (35.9)
	38-47	27 (26.5)	75 (73.5)	102 (24.9)
	48-57	35(38.0)	57 (62.0)	92 22.5)
	58-67	19 (44.2)	24 (55.8)	43 (10.5)
	67 and above	4 (30.8)	9 (69.2)	13 (3.2)
Sex	Male	69 (26.5)	191 (73.5)	260 (63.6)
	Female	29 (19.5)	120 (80.5)	149 (36.4)
Marital status	Single	9 (15.3)	50 (84.7)	59 (14.4)
	Married	75 (23.4)	246 (76.6)	321 (78.5
	Divorced / separated	3 (42.9)	4 (57.1)	7 (1.7)
	Widow /widower	11 (50.0)	11 (50.0)	22 (5.4)
Level of education	Never went to school	10 (32.3)	21 (67.7)	31 (7.6)
	Primary	12 (21.4)	44 (78.6)	56 (13.7)
	Secondary	11 (22.9)	37 (77.1)	48 (11.7)
	Certificate	2 (16.7)	10 (83.3)	12 (2.9)
	Diploma	2 (40.0)	3 (60.0)	5 (1.2)
	Bachelor's	17 (18.7)	74 (81.3)	91 (22.2)
	Master's	31 (21.8)	99 (76.2)	130 (31.8)
	PhD	9 (30.0)	21 (70.0)	30 (7.3)
	Post doctorate	4 (66.7)	2 (33.3)	6 (1.5)
Role at the University	Administrative staff	19 (20.4)	74 (79.6)	93 (22.7)
	Academic staff	45 (26.3)	126 (73.7)	171 (41.8
	Technician	8 (24.2)	25 (75.8)	33 (8.1)
	Support staff	26 (23.2)	86 (76.8)	112 (27.4)
Residence	Urban	94 (23.7)	302 (76.3	396 (96.8)
	Rural	4 (30.8)	9 (69.2)	13 (3.2)
Do you smoke?	Yes	4 (25.4)	22 (84.6)	26 (6.4)
	No	94 (24.5)	289 (75.5)	383 (93.6)
No of cigarettes per day	1 cigarette	0 (0)	3 (100)	3 (0.7)
	2 cigarettes	0 (0)	1 (100)	1 (0.2)
	3 cigarettes	0 (0)	5 (100)	5 (1.2)
	4 and more cigarettes	4 (23.5)	13 (76.5)	17 (4.2)
Do you drink alcohol?	Yes	23 (33.3)	46 (66.7)	69 (16.9)
	No	75 (22.1)	265 (77.9)	340 (83.1)
Units of alcohol per day	One unit	9 (30.0)	21 (70.0)	30 (7.3)
	Two units	7 (41.2)	10 (58.8)	17 (4.2)
	Three units	6 (54.5)	5 (45.5)	11 (2.7)
	Four units	1 (20)	4 (80.0)	5 (1.2)
	More than four units	0 (0)	6 (100)	6 (1.5)
Exercise	Twice daily	9 (20.7)	39 (81.3)	48 (11.7)
	Once daily	20 (23.9)	83 (74.1)	112 (27.4)
	Weekly	10 (18.5)	44 (81.5)	54 (13.2)
	Biweekly	13 (33.1)	24 (64.9)	37 (9.0)
	Thrice weekly	3 (16.7)	15 (83.3)	18 (4.4)
	Monthly	1 (6.7)	14 (93.3)	15 (3.7)
	Never at all	1 (50.0)	1 (50.0)	2 (0.5)
	Occasionally	32 (26.0)	91 (74.0)	123 (30.1)
Are you aware of your BP status?	Yes	59 (29.4)	142 (70.6)	201 (49.1)
	No	39 (18.7)	169 (81.3)	208 (50.9)
Have you ever screened for hypertension?	Yes	69 (30.0)	161 (70.0)	230 (56.2)
	No	29 (16.7)	150 (83.8)	179 (43.8)
How much sugar do you consume daily?	No sugar	14 (35.9)	25 (64.1)	39 (9.5)
	One teaspoon	15 (21.1)	56 (78.9)	71 (17.4)
	Two teaspoons	32 (38.6)	80 (71.4)	112 (27.4)
	More than two teaspoons	37 (19.8)	150 (80.2)	187 (45.7)
Are you diabetic?	Yes	15 (48.4)	18 (51.6)	31 (7.6)
	No	83 (22.0)	295 (78.0)	378 (92.4)
Is there any family history of hypertension??	Yes	28 (21.7)	101 (78.3)	129 (31.5)
	No	70 (25.0)	210 975)	280 (68.5)
Is there family history of diabetes?	Yes	24 (24.5)	74 (75.5)	98 (24.0)
	No	74 (23.8)	237 (76.2)	311 (76.0)
What is hypertension?	Increased BP above the normal	6 (25.9)7	192 (74.1)	259 (63.3)
	Decreased BP below the normal	0 (0)	4 (100)	4 (1.0)
	No idea	31 (21.2)	115 (78.8)	146 (35.7)
What is diabetes?	High blood sugar	61 (25.7	176 (74.3)	237 (57.9)
	Low blood sugar	3 (30,0)	7 (70.0)	10 (2.4)
	No idea	3 (21.0)4	128 (79.0)	162 (39.6)
Body mass index	Less than 18.5	0 (0)	18 (100)	18 (4.4)
	18.5 - 24.9	27 (15.3)	149 (84.7)	176 (43.0)
	25.0 - 29.9	51 (34.7)	96 (65.3)	147 (35.9)
	30 and above	20 (29.4)	48 (70.6)	68 (16.6)

**Table 2:** association between hypertension and risk factors

Parameters		OR 95% CI (LL, UL)	p-value	aOR 95% CI (LL, UL)	P-value
Age in years	18-37*				
	38-57	7.828 (3.595, 17.041)	<0.001	5.860 (2.507, 13.701)	<0.001
	≥ 58	1.484 (0.805, 2.736)	0.206	1.301 (0.664, 2.547)	0.443
Marital status	Single*				
	Married	5.556 (1.856, 16.63)	0.002	1.866 (0.534, 6.521)	0.329
	Divorced / separated	3.28 (1.368, 7.867)	0.008	2.415 (0.900, 6.478)	0.08
	Widow /widower	1.333 (0.240, 7.405)	0.742	0.774 (0.118, 5.098)	0.79
Alcohol consumption	No*				
	Yes	1.767 (1.007, 3.100)	0.047	2.198 (1.159, 4.171)	0.016
Awareness	Yes*				
	No	0.555 (0.350, 0.882)	0.013	0.719 (0.430, 1.203)	0.209
Diabetic	No*				
	Yes	3.332 (1.581, 7.021)	0.002	1.806 (0.785, 4.158)	0.164
Body mass index	≤ 24.9*				
	≥ 25.0	3.050 (1.857, 5.008)	<0.001	2.418 (1.412, 4.141)	0.001
* Reference category, test used: univariate logistic regression; OR: odds ratio and aOR: adjusted odds ratio; CI: confidence interval; LL: low limits, UL: upper limits.					

## Discussion

This study assessed the prevalence and associated risk factors of hypertension among the University of Juba staff. Based on the WHO definition of hypertension which considers an individual hypertensive if the average BP is greater or equal to 140/90 mm Hg, the prevalence of hypertension among the participants is 24% [9]. Significantly associated risk factors were age, alcohol consumption and increased body mass index. Comparatively, the prevalence in our study is lower than the prevalence (37%) observed among university employees in India [18]. It is also lower than the estimated global prevalence of 33.5% [19]. However, our result is higher than the results of a pioneer study conducted among potential blood donors in Juba, which reported a prevalence of 19.3% and those of a similar study among workers at Hawassa University, which had a lower prevalence of 19.7% [9,14]. In addition, while Sube and others showed a higher prevalence of 48.1% among diabetic patients attending diabetic retinopathy screen in Juba, lower prevalence (8.8%) was reported among university staff in Malaysia [15,20]. Comparable findings (25.4%) were reported in the African Region and in the findings (25.9%) of a multicountry study in sub-Saharan Africa (SSA) which examined hypertension among different population groups [3,21]. While these comparisons are being done across varying populations, one would argue that the settings are similar and there is a rising prevalence of hypertension in the country given the increment of 4.7% in our study compared to the reported pioneer study despite the use of two different population groups in these studies [14].

Why is hypertension rising? Our study documents the risk factors for hypertension as age, marital status, alcohol consumption, knowledge of hypertensive status, being diabetic and increased BMI. Demographic factors including age, location, marital status, and sex have been established to affect BP and are risk factors for hypertension [22]. In the present study, prevalence of hypertension was associated with advancing age of the employee. These findings are in agreement with several other studies including one involving Nigerian health service providers and another one conducted at Kenyatta University employees [23,24]. In rural Tanzania and Bangladesh, hypertension was 17% and 16% prevalent, respectively, lower than in our study [25,26]. The high prevalence of hypertension observed among university employees from rural settings (30.8%) in our study could suggest a previous predisposition to hypertension risk factors and further illustrates the influence of rural settings on the risk for increasing cases of hypertension. Rural environments may be marked by availability of food and dietary habits that contribute to weight gain and increased susceptibility to obesity as well as insecurity stress level, factors closely linked to the risk of hypertension. In the current study, academic staff were more hypertensive than their nonacademic counterparts. This concurs with Bosu (2016) and Asresahegn *et al*. (2017) who showed that those who had higher levels of education were three times more likely to be affected by hypertension than those who had lower qualifications [27,28].

In this study, a BMI ≥ 25 was associated with high BP, an observation that agrees with previous studies [28-30]. It is worth noting that obesity increases the risk of developing hypertension and other cardiovascular disorders and this has been demonstrated in this study, where obese participants (29.4%) are twice as likely to be hypertensive. In this study only 24.7% practiced physical exercise despite the fact that physical inactivity is associated with a high risk of increased deposition of body fat, overweight and obesity with subsequent cardiometabolic complications [31]. An individual´s decision and willingness to engage in physical exercise may be influenced by factors such as infrastructural challenges and lack of time [32,33]. Our study did not examine these relationships. We also found that alcohol consumption was significantly associated with hypertension and was a bit higher than the reported consumption from neighboring Uganda [34]. In addition, being male was a significant driver of alcohol consumption in line with the results established by Bjertness *et al*. [35]. This could explain why male participants were three times more likely to have hypertension than their female counterparts. This relationship seems to be well established by several studies [36-39]. This association has been explained by other high-risk habits among men, such as social aggregation and smoking and adding salt to food while eating [40]. No significant relationship was found between hypertensive status and variables such as exercise, sex, marital status, smoking, educational level, residents, and sugar consumption. Family history of hypertension and family history of diabetes were not significant according to our study.

These findings are rather contradictory in some cases. Several studies have reported many of the risk factors, which are not significant in our study. For instance, family history of hypertension or cardiovascular diseases and low physical activity were associated with hypertension among Chinese and Swedish men and women [7]. Similarly, low physical activity, unhealthy diet, dyslipidaemia, history of smokers and feeling accounted for hypertension in Saudi Arabia [8]. Perhaps further studies using larger populations could explain these variations. Given the economic burden of hypertension, it is likely to be a daunting task for countries, such as South Sudan, to tackle the disease when it gets higher than this. Assuming these results were to be generalized to the whole country, 24% is approximately one in every four people, and this would be close to two million hypertensive South Sudanese, which would attract a huge cost! Also, 28.6% have average BP above the threshold of normotensive (120/ 80mmHg or less). These are candidates for hypertension in the long run if no preventive measures are taken. Worse still, combining this group with the hypertensive ones translates to more than half of the respondents being above the normal threshold! This is something to ponder in light of the seemingly rapid epidemiological transition in the country. Given that this study was carried out in one institution only, generalization of the results to the whole country should be made with caution, as this cannot be guaranteed. Secondly, it was a cross-sectional study and therefore has not established any temporal relationships between the independent and dependent variables. Also, important to note is the inability to include risk factors such as lipid profile due to the costs involved. The findings rely on individual reports, which may be affected by factors beyond the scope of this study. On a good note, this study is among the first of its kind in the country, and it is the first to be conducted among the university staff. It is therefore a harbinger in the provision of evidence on the status of hypertension in the country. In addition, robust scientific methods and research principles were followed rigorously to ensure accurate and sound evidence is generated.

## Conclusion

Close to a quarter of the University of Juba staff is hypertensive. Age, alcohol consumption and increased body mass index were significantly associated with the disease. In light of previous studies in the country there is a rise in the prevalence of the disease. The study recommends tailored preventive interventions against hypertension at the University.

### What is known about this topic

- Some studies report a high prevalence of non-communicable diseases among some segments of the population in South Sudan;
- Unpublished sources show a rising burden of NCDs in the country;
- Very limited studies have been conducted in the country, and none was conducted among university staff.

### What this study adds

- There is a high prevalence of hypertension among the University of Juba staff;
- Risk factors including age, marital status, obesity, alcohol consumption, high body mass index, and being diabetic are predictors of hypertension among the study population;
- A very low level of awareness about hypertension was documented in the study.
